# Regulation of Petrobactin and Bacillibactin Biosynthesis in *Bacillus anthracis* under Iron and Oxygen Variation

**DOI:** 10.1371/journal.pone.0020777

**Published:** 2011-06-06

**Authors:** Jung Yeop Lee, Karla D. Passalacqua, Philip C. Hanna, David H. Sherman

**Affiliations:** 1 Life Sciences Institute and Departments of Medicinal Chemistry, Chemistry, Microbiology and Immunology, University of Michigan, Ann Arbor, Michigan, United States of America; 2 Department of Microbiology and Immunology, University of Michigan Medical School, Ann Arbor, Michigan, United States of America; Baylor College of Medicine, United States of America

## Abstract

**Background:**

*Bacillus anthracis* produces two catecholate siderophores, petrobactin and bacillibactin, under iron-limited conditions. Here, we investigate how variable iron and oxygen concentrations influence the biosynthetic output of both siderophores in *B. anthracis*. In addition, we describe the differential levels of transcription of select genes within the *B. anthracis* siderophore biosynthetic operons that are responsible for synthesis of petrobactin and bacillibactin, during variable growth conditions.

**Methodology/Principal Findings:**

Accumulation of bacillibactin in *B. anthracis* Sterne (34F_2_) and in a mutant lacking the major superoxide dismutase (*ΔsodA1*) was almost completely repressed by the addition of 20 µM of iron. In contrast, petrobactin synthesis in both strains continued up to 20 µM of iron. Accumulation of petrobactin and bacillibactin showed a slight increase with addition of low levels of paraquat-induced oxidative stress in wild type *B. anthracis* Sterne. Cultures grown with high aeration resulted in greater accumulation of petrobactin relative to low aeration cultures, and delayed the repressive effect of added iron. Conversely, iron-depleted cultures grown with low aeration resulted in increased levels of bacillibactin. No difference was found in overall superoxide dismutase (SOD) activity or transcriptional levels of the *sodA1* and *sodA2* genes between iron-depleted and iron-replete conditions at high or low aeration, suggesting that SOD regulation and iron metabolism are separate in *B. anthracis*. The highest transcription of the gene *asbB,* part of the petrobactin biosynthetic operon, occurred under iron-limitation with high aeration, but transcription was readily detectable even under iron-replete conditions and in low aeration. The gene *dhbC*, a member of the bacillibactin biosynthetic operon, was only transcribed under conditions of iron-depletion, regardless of growth aeration.

**Conclusion:**

These data suggest that bacillibactin regulation is highly sensitive to iron-concentration. In contrast, although regulation of petrobactin is less dependent on iron, it is likely subject to additional levels of regulation that may contribute to virulence of *B. anthracis*.

## Introduction

Iron acquisition is a crucial process for most organisms since this element is an essential component of enzymes of primary metabolism [1]. Most living organisms require iron as a cofactor for enzymes that are involved in redox reactions and other essential functions and possess efficient systems for the uptake, transport and storage of this element [1], [2]. For bacteria, acquisition of iron is facilitated by high affinity metal chelators called siderophores that are actively taken up by iron-depleted cells through specific membrane receptors [1], [2], [3], [4]. Although iron has an essential role in cell metabolism, free iron can be deleterious to cells by forming oxygen radicals after reaction with hydrogen peroxide. Consequently, iron acquisition and storage in microorganisms must be efficiently regulated to avoid both starvation and toxicity.

Iron and oxygen are essential for aerobic life, but oxygen-derived species such as superoxide and hydrogen peroxide that occur as by-products of aerobic metabolism, are potentially toxic [5]. To face this threat, all organisms have defense mechanisms against oxygen derivatives and also tightly regulate the intracellular iron concentration [2], [4], [6]. Evidence has started to emerge for coordination between the expression of antioxidant enzymes and iron metabolism (transport, sequestration). The regulation of superoxide dismutase (SOD) in *E. coli* illustrates this coordination. SOD is a ubiquitous enzyme that scavenges intracellular superoxide radicals, playing a major role in the defense system against oxidative stress [7], [8]. Thus, either increased generation of superoxide or an increase in steady state levels of superoxide can create oxidative stress.

**Figure 1:**
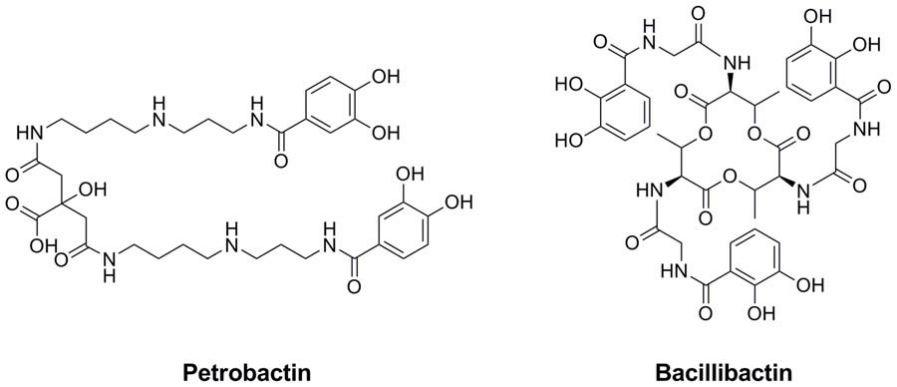
Chemical structures of *B. anthracis* siderophores petrobactin and bacillibactin.

*B. anthracis* is a zoonotic soil microorganism and the causative agent of anthrax. This bacterium is a highly virulent mammalian pathogen and among the known biological-warfare agents. *B. anthracis* is widely accepted as one of the most serious threats because of the resilience of its spores and potential use as a bioweapon [9], [10]. *B. anthracis* produces two catecholate siderophores, petrobactin and bacillibactin (Figure 1), under iron-limited conditions [11] and the biosynthesis of these molecules is associated with two operons, *asb* (for petrobactin) and *bac* (for bacillibactin). It has been demonstrated that only the *asb* locus is required for growth in iron-depleted media and for virulence in a mouse model [11].

In previous work [12], we described the first details on the biosynthesis of petrobactin by identification of its biosynthetic intermediates isolated from various *B. anthracis asb* mutants. In addition, we provided the basis for assigning the order of assembly and presumed role for gene products encoded by the *asb* operon [13]. Previous studies also revealed the four putative *sod*s encoded on the *B. anthracis* genome, and *sodA1* is the main paralog responsible for protection from endogenous superoxide stress [14]. Additionally, wild type *B. anthracis* Sterne and the *ΔsodA1* mutant displayed a major increase in the transcription of genes involved in bacillibactin biosynthesis and iron/metal transport after treatment with paraquat (800 µM) [15]. However, actual levels of both bacillibactin and petrobactin were decreased after treatment with 800 µM paraquat [15]. Therefore, we sought to further characterize the relationship between siderophore production and milder forms of oxidative stress in both wild type and *ΔsodA1* mutant of *B. anthracis.*

In this study, experiments were conducted to investigate how variable iron concentrations, low levels of the redox cycling compound paraquat, and differing levels of atmospheric oxygen could influence the biosynthetic output of both the *B. anthracis* siderophores petrobactin and bacillibactin. In addition, we describe the differential levels of transcription of select genes within the *B. anthracis* siderophore biosynthetic operons that are responsible for assembly of petrobactin and bacillibactin, during variable growth conditions. The *B. anthracis ΔsodA1* mutant, which is attenuated for growth in metal-chelated medium, was used in addition to wild type Sterne to determine if siderophore-mediated iron metabolism and SOD activity are linked in this pathogenic microorganism.

## Results and Discussion

### Iron Concentration Represses Accumulation of *B. anthracis* Siderophores Petrobactin and Bacillibactin

In general, the biosynthesis of siderophores is negatively regulated by iron [1] and this has been demonstrated in many microorganisms [16], [17], [18]. In iron-limited cultures of *Azotobacter vinelandii*, the siderophore azotobactin was present in a 1 µM iron-fed medium and was completely repressed at 3 µM iron [17]. In the fungus *Ustilago maydis*, when iron was limiting in the growth medium, it produced copious amounts of the cyclic peptide siderophores ferrichrome and ferrichrome A [16]. However, accumulation of siderophores was almost completely eliminated within 6 hours after the addition of iron to cultures of the fungus. Addition of 7 µM iron to the iron-limiting cultures of *Fusarium venenatum* A3/5 almost completely repressed siderophore production [18]. Therefore, we sought to investigate how overall accumulation of both siderophores petrobactin and bacillibactin in *B. anthracis* changes by the addition of iron to iron-limited cultures.

We compared accumulation of petrobactin and bacillibactin in iron-depleted medium (IDM) cultures of *B. anthracis* wild type Sterne (34F_2_), the mutant *ΔsodA1*, and *ΔasbABCDEF* and *ΔdhbACEBF* (as negative controls for the production of petrobactin and bacillibactin, respectively) that were grown for 24 hours under iron-depleted (no addition of Fe) and iron-replete (addition of 3, 6, 10 and 20 µM Fe) conditions. Quantities of petrobactin and bacillibactin from *B. anthracis* were monitored using LCMS after isolating from supernatants of 24 hour cultures of each strain in IDM with the indicated levels of iron-supplementation (Table 1). The LCMS analysis revealed that in iron-depleted conditions, higher levels of petrobactin and bacillibactin accumulated in wild type Sterne than in the mutant *ΔsodA1* (almost 2-fold and 4-fold amounts of petrobactin and bacillibactin, respectively) (Table 1). Overall, when iron was added to the iron-depleted cultures, the amount of petrobactin accumulated was more in *ΔsodA1* than Sterne. However, there was no significant difference in the amount of bacillibactin accumulated between Sterne and *ΔsodA1* after iron addition, where bacillibactin is barely detectable when even 3 µM iron was present. As anticipated, neither petrobactin in *ΔasbABCDEF* nor bacillibactin in *ΔdhbACEBF* was detected under any conditions (data not shown).

**Table 1:** Quantification of the petrobactin and bacillibactin siderophores produced by *Bacillus anthracis* Sterne 34F_2_ and the mutant Δ*sodA1* under IDM cultures with the addition of different concentrations of iron.

Iron (µM)	Petrobactin (µg/ml) a		Bacillibactin (µg/ml) a	
	Sterne 34F_2_	Δ*sodA1*	Sterne 34F_2_	Δ*sodA1*
0	132.430±11.035 b	66.609±9.341	1.362±0.023	0.283±0.005
3	24.046±5.678	65.789±7.451	0.004±0.000	0.003±0.001
6	20.486±5.101	39.905±6.450	0.003±0.000	0.003±0.000
10	18.888±4.789	25.623±4.346	0.002±0.000	0.003±0.000
20	17.035±4.456	25.209±4.509	0.001±0.000	0.001±0.000

a Concentration of siderophores petrobactin and bacillibactin is expressed as µg/ml of supernatant from IDM cultures of *B. anthracis*.

b Quantification was performed three times from the independent extraction and values represent means ± standard deviations.

**Figure 2:**
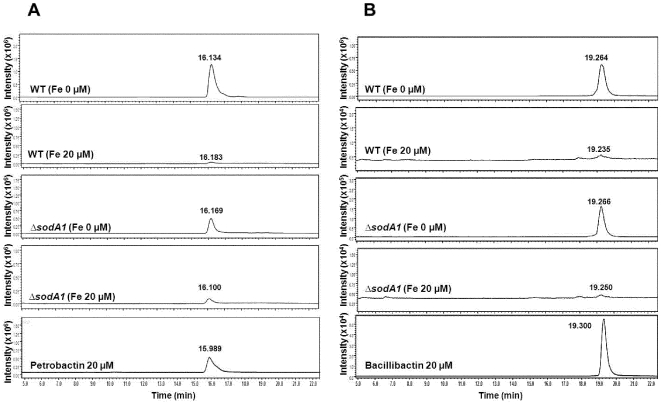
LCMS traces of extracts from the IDM cultures of *B. anthracis* showing the presence of petrobactin and bacillibactin with or without addition of iron. (A) Selected ion monitoring (SIM) chromatogram of petrobactin at the mass range of *m/z* 719.3 [M+H]^+^. The peaks at 15.989–16.183 min represent petrobactin. (B) SIM chromatogram of bacillibactin at the mass range of *m/z* 883.2 [M+H]^+^. The peaks at 19.235–19.300 min represent bacillibactin.

The general pattern in *B. anthracis* showed that as the iron concentration increased, the accumulation of both siderophores decreased (Table 1 and Figure 2). The abolished production of bacillibactin in Sterne and *ΔsodA1* in medium with 3 µM iron and the barely detectable levels of this siderophore in 20 µM iron is comparable to what has been observed in *A. vinelandii* [17], and the fungi *Rhodotorula mucilaginosa* [19] and *Rhizopus microsporus* [20], where the production of azotobactin and fungal siderophores were repressed under similar conditions. However, petrobactin synthesis in both Sterne and *ΔsodA1* occurred up to 20 µM of iron. Taken together, these data suggest that there might be additional factors involved in the regulation of petrobactin synthesis in *B. anthracis* in addition to the ferric uptake repressor (Fur), and that regulation of the biosynthesis of this particular metabolite is not entirely controlled by iron concentration.

### Low Levels of Paraquat Induce Slight Increases in Accumulation of Petrobactin and Bacillibactin

Paraquat is an oxygen radical generator and it is known to induce catalase and superoxide dismutase activities, presumably as oxidative stress protection mechanisms in the microbial cells [21], [22]. Previously, we showed that high levels of paraquat stress (100 µM and 800 µM) increases the transcription of genes involved in bacillibactin biosynthesis, but that these levels of paraquat decrease end-point bacillibactin and petrobactin accumulation [15]. We therefore, reasoned that major intracellular redox stress at these levels may interfere with the redox sensitive enzymatic steps of siderophore biosynthesis, and that lower, more biological levels of oxidative stress may increase siderophore production.

To investigate this, wild type Sterne and the mutant *ΔsodA1* were exposed to several mild concentrations of paraquat (0, 5, 15, 25 and 50 µM) in IDM. Note that in wild type cells, 800 µM paraquat is non-toxic, and thus the low paraquat concentrations used here are presumably promoting very low-levels of endogenous superoxide stress. Quantities of petrobactin and bacillibactin from the paraquat-exposed *B. anthracis* were analyzed using LCMS by isolating 24 hour supernatant cultures of the strains in IDM (Table 2). It should be noted that when spores of *B. anthracis* strains Sterne and *ΔsodA1* were used to inoculate IDM cultures with paraquat already added, no growth was observed, even under the lowest concentration of paraquat (5 µM; data not shown), which indicates that paraquat is very toxic to spore germination when iron is limiting. Therefore, cultures were grown to saturation and these vegetative cultures were back-diluted into fresh IDM medium with the indicated amount of paraquat present.

**Table 2:** Quantification of the *Bacillus anthracis* siderophores petrobactin and bacillibactin obtained from the IDM cultures of the strains Sterne 34F_2_ and Δ*sodA1* with the addition of different concentrations of paraquat.

Paraquat (µM)	Petrobactin (µg/ml) a		Bacillibactin (µg/ml) a	
	Sterne 34F_2_	Δ*sodA1*	Sterne 34F_2_	Δ*sodA1*
0	103.432±9.300 b	61.883±5.430	0.179±0.038	0.632±0.135
5	104.731±13.456	34.537±5.010	0.823±0.101	0.001±0.000
15	108.623±7.891	22.785±3.214	1.434±0.231	NO c
25	120.907±11.235	20.966±3.010	1.935±0.201	NO
50	125.701±14.500	12.358±2.340	2.026±0.130	NO

a Concentration of siderophores petrobactin and bacillibactin is expressed as µg/ml of supernatant from IDM cultures of *B. anthracis*.

b Quantification was performed three times from the independent extraction and values represent means ± standard deviations.

c NO, not observed.

*Paraquat (PQ) concentration and incubation times before cell lysate and supernatant harvesting for siderophore quantification are as follows: For exponentially growing cells, 800 µM paraquat was added at OD_600_ 0.4–0.5 and cells were harvested after 1 hour.

**Note that of all the samples, the growth of *ΔsodA1* is slowed by the addition of paraquat, and so the final OD_600_ of this sample was slightly lower than that of all others.

**Figure 3:**
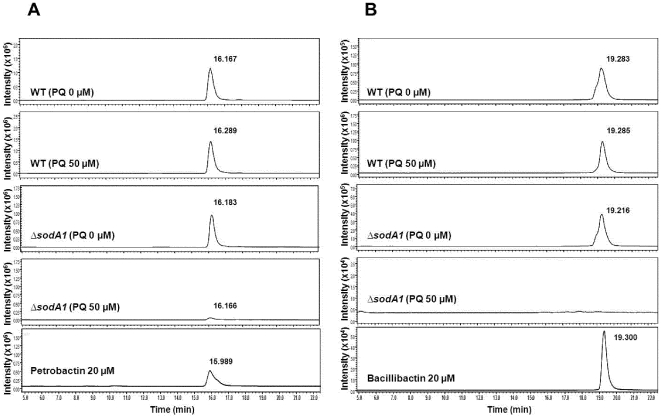
LCMS chromatogram of petrobactin and bacillibactin in the extracts obtained from the IDM cultures of *B. anthracis* with or without addition of paraquat. (A) SIM chromatogram of petrobactin at the mass range of *m/z* 719.3 [M+H]^+^. The peaks at 15.989–16.289 min represent petrobactin. (B) SIM chromatogram of bacillibactin at the mass range of *m/z* 883.2 [M+H]^+^. The peaks at 19.216–19.300 min represent bacillibactin.

LCMS analysis showed that under iron-limited conditions, accumulation of petrobactin and bacillibactin were slightly increased by addition of paraquat in *B. anthracis* wild type Sterne (Table 2 and Figure 3). This strain responded to paraquat treatment by a gradual increase in accumulations of petrobactin and bacillibactin at each concentration and showed 1.2-fold and 11.9-fold increase in petrobactin and bacillibactin, respectively, at higher concentration of 50 µM paraquat. However, for the *B. anthracis ΔsodA1* mutant, a significant decrease in accumulation of petrobactin and bacillibactin was observed dependent upon the treatment with paraquat, indicating a higher sensitivity of iron-starved cells of *ΔsodA1* toward paraquat treatment. It is notable that accumulation of bacillibactin was almost completely repressed by the addition of paraquat in *ΔsodA1* (700-fold-lower level compared to bacillibactin content in wild type Sterne at 5 µM paraquat) and no bacillibactin was detected at the concentration of more than 15 µM paraquat, due mainly to the strong deleterious effect of paraquat on growth of this mutant in iron-depleted conditions, underscoring the importance of *sodA1* to *B. anthracis* for protection from endogenous superoxide stress, particularly when iron is limiting. Interestingly, despite the severe growth defect experienced by *ΔsodA1* in IDM with paraquat, petrobactin were still detected in these cultures. However, unlike Sterne, petrobactin levels decrease as paraquat increases, suggesting that in addition to slowed growth, the biosynthetic enzymes for petrobactin may be highly sensitive to redox conditions, and that *sodA1* is needed to maintain suitable intracellular redox conditions.

Previous studies have shown that paraquat decreased accumulation of the siderophore pyoverdine in iron-limited cultures of *Pseudomonas aeruginosa* [23]. In contrast, Eisendle and co-workers [24] have demonstrated that in the fungus *Aspergillus nidulans*, during iron starvation, accumulation of the siderophore ferricrocin was upregulated by the addition of oxidative stress agent paraquat. Tindale et al. [17] reported up-regulation of *csbC*, an early gene required for the catecholate siderophore biosynthesis, which encodes an isochorismate synthase in *A. vinelandii* by the addition of paraquat. Similarly, our data show that oxidative stress causes slightly increased, or at least steady state levels of accumulation of both petrobactin and bacillibactin for *B. anthracis* wild type Sterne during iron-depleted conditions.

### Iron-Related Repression of Petrobactin Is Delayed by High Aeration and Bacillibactin Biosynthesis Is Promoted during Low Aeration

Previously, Cendrowski *et al.* [11] identified two operons encoding the enzymes responsible for biosynthesis of the two *B. anthracis* siderophores, named *asb* and *bac*, which are responsible for the biosynthesis of petrobactin and bacillibactin, respectively. To investigate a possible effect of oxygen tension on the regulation of the *asb* and *bac* operons that may result in changes in accumulation of petrobactin and bacillibactin under iron-limited conditions, cultures were grown for 24 hours in IDM with variable concentrations of iron, and aeration altered by varying culture volume per flask. Quantities of petrobactin and bacillibactin were monitored using LCMS as described in Materials and Methods.

**Figure 4:**
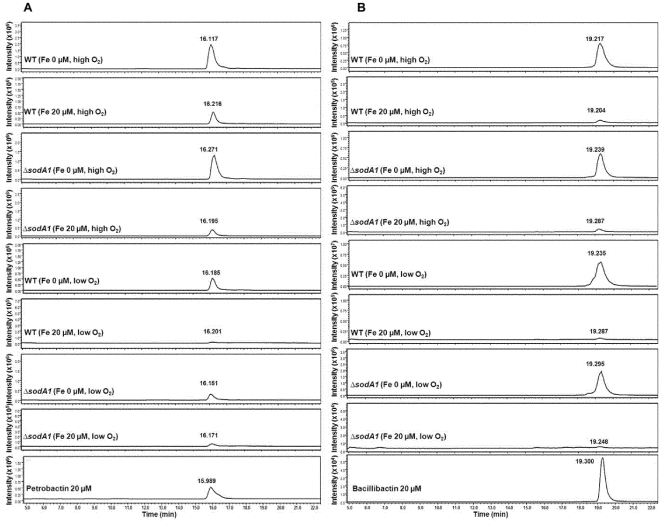
LCMS profiles of petrobactin and bacillibactin from the extracts of the IDM cultures of *B. anthracis* with or without addition of iron under high and low aeration. (A) SIM chromatogram of petrobactin at the mass range of *m/z* 719.3 [M+H]^+^. The peaks at 15.989–16.271 min represent petrobactin. (B) SIM chromatogram of bacillibactin at the mass range of *m/z* 883.2 [M+H]^+^. The peaks at 19.204–19.300 min represent bacillibactin.

The addition of iron to the IDM cultures of *B. anthracis* caused the repression of accumulation of petrobactin with both high and low aeration (Table 3 and Figure 4A). As described earlier in this study (Table 1), petrobactin accumulation in both strains continued up to a concentration of 20 µM iron regardless of high or low aeration. However, higher accumulation of petrobactin was observed in both Sterne and the mutant *ΔsodA1* in the high aeration cultures relative to the low aeration samples (Table 3) at all iron-concentrations, indicating that increased aeration promotes the biosynthesis of petrobactin and delays the repressive effect of added iron.

**Table 3:** Quantification of petrobactin from IDM cultures of *Bacillus anthracis* Sterne 34F_2_ and Δ*sodA1* with the addition of different concentrations of iron under high (high O_2_) and low aeration (low O_2_).

Iron (µM)	Petrobactin (µg/ml) a under high O_2_		Petrobactin (µg/ml) under low O_2_	
	Sterne 34F_2_	Δ*sodA1*	Sterne 34F_2_	Δ*sodA1*
0	193.764±13.012 b	135.838±9.089	55.669±4.502	40.973±3.450
3	96.988±9.345	100.247±10.341	23.188±4.568	24.646±2.310
6	84.372±10.101	86.371±5.680	21.418±3.213	23.882±4.101
10	80.333±5.670	65.322±4.510	19.610±3.217	22.869±1.120
20	51.402±3.401	43.005±5.101	19.495±1.101	17.863±2.310

a Concentration of petrobactin is expressed as µg/ml of supernatant from IDM cultures of *B. anthracis*.

b Quantification was performed three times from the independent extraction and values represent means ± standard deviations.

The accumulation of bacillibactin was severely repressed by addition of iron to IDM cultures of both Sterne and *ΔsodA1* with high and low aeration (Table 4 and Figure 4B). Surprisingly, counter to the case for petrobactin, the highest levels of bacillibactin in Sterne and *ΔsodA1* were found in IDM (no iron supplementation) under low aeration. Koppisch et al. [25] reported that relatively low levels of bacillibactin accumulated in iron-depleted cultures of *B. anthracis*, but they conversely observed that cultures grown in ambient air seemed to produce higher levels of bacillibactin. However, we found that IDM cultures grown in a 40% culture-to-flask volume ratio resulted in the highest levels of bacillibactin we have seen thus far (average ∼ 16 µg/ml in low aeration versus 2 µg/ml in high aeration for Sterne, the latter representing an 80% decrease, and the measurement being near our detection limits). Hence, whereas high aeration with iron-depletion promotes the accumulation of petrobactin, the opposite condition of low aeration with iron-depletion promotes the accumulation of bacillibactin. These data support the idea that petrobactin and bacillibactin production are not subject to the same levels of regulation, and suggest that metal acquisition systems have evolved differently in *B. anthracis* compared to its relative *B. subtilis* [26], where *B. subtilis* relies primarily on bacillibactin for iron scavenging. Although *B. subtilis* is a model organism for the study of Gram-positive bacteria, *B. anthracis* has evolved a pathogenic lifestyle, exploiting non-soil growth environments, and revealing variety in the evolution of metal-acquisition regulation.

**Table 4:** Quantification of bacillibactin from IDM cultures of *Bacillus anthracis* Sterne 34F_2_ and Δ*sodA1* with the addition of different concentrations of iron under high (high O_2_) and low aeration (low O_2_).

Iron (µM)	Bacillibactin (µg/ml) a under high O_2_		Bacillibactin (µg/ml) under low O_2_	
	Sterne 34F_2_	Δ*sodA1*	Sterne 34F_2_	Δ*sodA1*
0	2.266±0.350 b	1.431±0.092	16.561±2.345	4.092±0.769
3	1.189±0.105	0.243±0.001	0.149±0.004	0.043±0.049
6	0.203±0.110	0.224±0.010	0.097±0.001	0.008±0.001
10	0.072±0.003	0.008±0.001	0.017±0.003	0.004±0.001
20	0.039±0.001	0.004±0.001	0.004±0.001	0.003±0.001

a Concentration of bacillibactin is expressed as µg/ml of supernatant from IDM cultures of *B. anthracis*.

b Quantification was performed three times from the independent extraction and values represent means ± standard deviations.

### Superoxide Dismutase (SOD) Activity from Whole Cell Lysates of *B. anthracis* Grown in IDM with Varying Aeration

**Figure 5:**
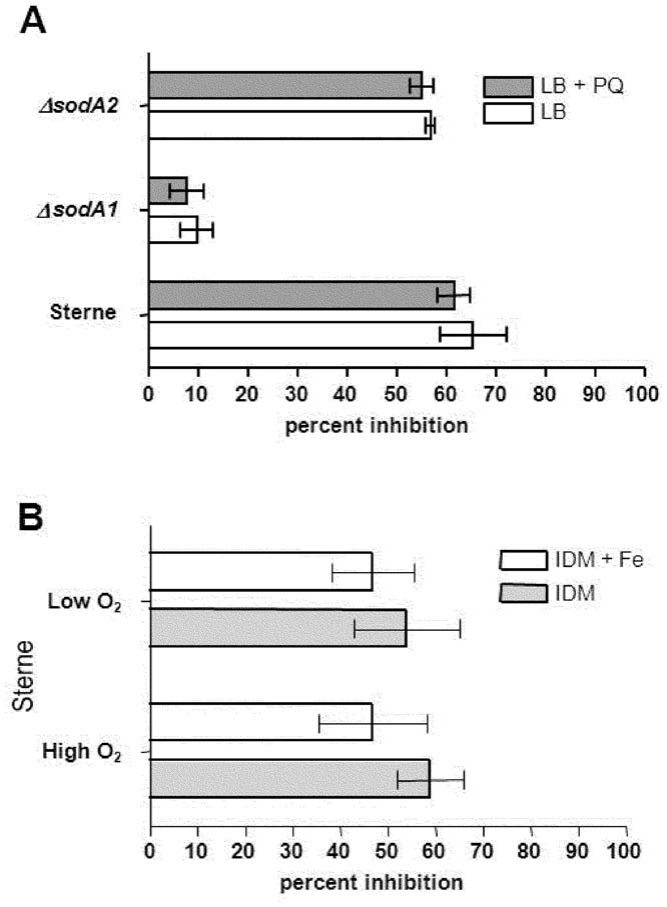
Nitroblue tetrazolium salt (NBT) assay of superoxide dismutase (SOD) activity from whole cell lysates of *B. anthracis* grown in various growth conditions. (A) Percent inhibition of superoxide production from 5 µg of whole cell lysates of *B. anthracis* Sterne 34F_2_, and the mutants *ΔsodA1* and *ΔsodA2* grown in LB (iron-rich) medium with and without the addition of a high concentration of paraquat (800 µM). (B) Percent inhibition of superoxide production from 5 µg of whole cell lysates of *B. anthracis* Sterne 34F_2_ grown in IDM and IDM supplemented with 20 µM Fe(II)SO_4_•7H_2_O under high and low aeration. Low O_2_ indicates cultures grown in a 40% culture to flask volume/volume ratio with slow shaking (125 rpm); High O_2_ indicates cultures grown in a 10% culture to flask volume/volume ratio with rigorous shaking (300 rpm). Percent inhibition indicates the amount by which the cell lysate inhibits NBT reduction relative to a non-lysate containing control reaction (see Materials and Methods).

Previous work has shown that overall SOD enzymatic activity in the microorganism *Azotobacter vinelandii* increases with increasing iron concentration [17]. Additionally, in *E. coli*, a connection between iron, oxygen and SOD regulation has been investigated [27], [28], [29]. Therefore, we sought to explore whether overall SOD enzymatic activity in *B. anthracis* changes between iron depleted and iron-replete conditions as it does in *A. vinelandii* [17], because iron-regulated gene expression has been observed to change during oxidative stress in *B. anthracis* [15]. It has been shown that two of the four putative SODs (SODA1 and SODA2) of *B. anthracis* can be isolated in active form, but that only *sodA1* is necessary for resistance to endogenous superoxide stress [14]. Figure 5A shows the results of a Nitroblue Tetrazolium Salt assay of superoxide dismutase activity (NBT-SOD assay) from whole cell lysates of wild type Sterne and the mutants *ΔsodA1* and *ΔsodA2* grown in LB medium, with and without the addition of the redox cycling reagent paraquat (800 µM). The overall SOD activity in Sterne did not change significantly after paraquat induced oxidative stress. The *ΔsodA1* mutant under both conditions revealed a severe reduction of SOD activity, whereas the *ΔsodA2* mutant showed almost identical overall SOD activity as wild type Sterne in these growth conditions. These results demonstrate that SODA1 is the primary superoxide dismutase in *B. anthracis.*

Figure 5B shows the results of Sterne cells grown in iron-depleted conditions [30] with and without iron supplementation and under high and low aeration. Overall, there are no significant differences in the levels of SOD activity with or without added iron, or at high or low aeration. Non-denaturing PAGE assays of SOD activity from these lysates revealed that SODA1 is the paralog predominantly present under iron-limitation with both high and low aeration (not shown). These data suggest that unlike *A. vinelandii* [17], there is no difference in overall superoxide scavenging ability of *B. anthracis* between iron-depleted and iron-replete conditions at high or low aeration. Other metal and oxidative conditions that may affect overall SOD activity in this organism are currently under investigation.

### SYBR-Green Quantitative RT-PCR of Select Genes from the *Asb* and *Bac* Operons in Iron-Depleted and Iron-Replete Conditions with Varying Aeration

**Figure 6:**
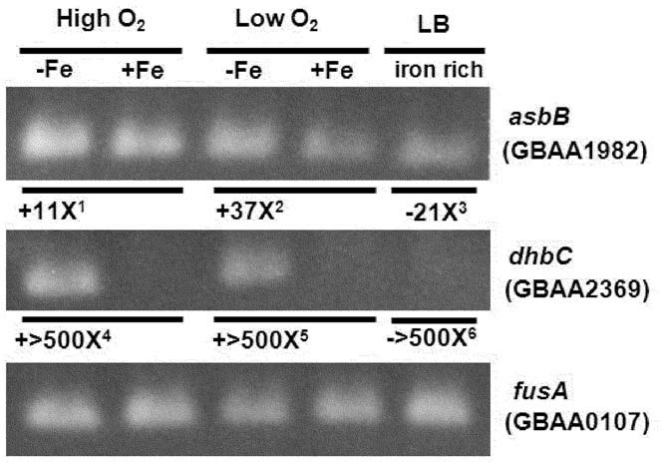
End-Point RT-PCR and SYBR-Green qRT-PCR of select genes from the *asb* and *bac* operons in iron-depleted, iron-supplemented, and iron-rich conditions. Ethidium bromide stained agarose gels from endpoint RT-PCR of the second gene in the *asb* operon (*asbB*), the second gene in the *bac* operon (*dhbC*) and a constitutively expressed elongation factor (*fusA*) from *B. anthracis* Sterne 34F_2_ grown in different media with various iron concentrations. –Fe is Iron-Depleted Medium (IDM); +Fe is IDM with 20 µM Fe(II)SO_4_ supplementation; LB is Luria Bertani Broth (∼7.6 µM iron concentration). High O_2_ and low O_2_ are cultures grown in high or low aeration respectively. LB cultures were grown under high O_2_ conditions (see Methods). Numbers are approximations of fold differences in transcript abundance as assayed by SYBR-Green qRT-PCR representing the following comparisons: ^1^ *asbB* is ∼11-fold more highly expressed in IDM with high aeration than in IDM+Fe with high aeration. ^2^ *asbB* is ∼37 fold more highly expressed in IDM with low aeration than in IDM+Fe with low aeration. ^3^ *asbB* is ∼21 fold less abundant in LB medium than in IDM with high aeration. Note that there is less than a 2-fold difference in transcript abundance between LB and IDM+Fe. ^4^ *dhbC* is greater than 500 fold more highly expressed in IDM with high aeration than in IDM+Fe with high aeration. ^5^ *dhbC* is greater than 500 fold more highly expressed in IDM with low aeration than in IDM+Fe with low aeration. ^6^ *dhbC* is more than 500 fold less abundant in LB medium than in IDM with high aeration. Note that the abundance of transcript in LB medium as compared to IDM+Fe is approximately the same.

Previously, it was shown that a *B. anthracis* mutant lacking the biosynthetic operon for petrobactin synthesis (*asb* operon) is attenuated for growth in iron-depleted medium, but is capable of eventual entry into stationary phase at the same level as wild type [12]. Because this mutant has an intact *bac* operon, it is possible that the bacillibactin siderophore is able to support growth in iron-poor conditions in place of petrobactin. Because *B. anthracis* produces two unique siderophores, one of which (petrobactin) appears to play a more predominant role in overall growth and physiology, we decided to assess transcriptional levels of select genes from the *asb* and *bac* loci in iron-rich and iron-poor growth conditions. Figure 6 shows the results of SYBR-Green quantitative RT-PCR (qRT-PCR, numeric data) and end-point RT-PCR (gels) of two early pathway genes in the *asb* and *bac* operons (*asbB* and *dhbC*, respectively). First, mRNA for the *asbB* gene was detected during growth in rich (LB), iron-poor (-Fe: IDM alone), and iron-supplemented (+Fe: IDM +20 µM Fe(II)SO_4_) conditions. The *dhbC* gene, however, was only detected in cells grown in IDM with no iron supplementation (both aeration conditions). The abrogation of transcription of *dhbC* in the presence of iron suggests that bacillibactin biosynthesis is tightly regulated by iron concentration at the transcriptional level.

Closer inspection of the quantitative differences in gene expression for *asbB* revealed variant expression patterns for this gene between iron-depleted (−Fe) and iron-supplemented (+Fe) conditions. In both high (↑O_2_) and low aeration (↓O_2_), the *asbB* gene was more highly expressed when iron was limited (11-fold and 37-fold higher expression, respectively). In addition, comparing the amount of *asbB* expression between cells grown in rich medium versus in IDM (−Fe) with ↑O_2_, *asbB* transcript is about 21-fold less abundant in the rich medium. Finally, the amount of *asbB* expression in IDM with iron added under high and low aeration is equivalent. Collectively, these data suggest that although the highest transcription of *asbB* occurs under iron-limitation, transcription is not effectively shut down when iron is present, as is the case for *dhbC*. The presence of oxygen appears to support more active transcription of *asbB* than when aeration is low, but in effect, this gene transcript was detectable in all conditions tested. These transcriptional data mirror the results of petrobactin and bacillibactin metabolite accumulation described here, where higher levels of petrobactin are isolated during high aeration, but accumulation of this metabolite is not abolished by iron supplementation as found with bacillibactin. Other transcriptome studies have shown that the *asb* operon, in its entirety, is detected under multiple growth conditions [31], [32], where the lowest levels of transcription occurred in the sporulation phase. These data suggest that the bacillibactin locus is highly regulated by the presence of iron, whereas the petrobactin locus is only mildly affected at the transcriptional level by cellular iron concentrations, and is more constitutive in nature. The *B. anthracis* genome contains several genes for multiple Fur-like regulatory proteins, and future work will focus on the biochemical roles that these proteins play in the regulation of these two siderophore operons.

*B. anthracis* utilizes secreted hemophores to scavenge heme from host hemoglobin, thereby facilitating iron acquisition from extracellular heme pools and delivering it to iron-regulated surface determinant (Isd) proteins [33], [34]. A recent transcriptome study [35] showed that the iron acquisition system related to these proteins is important for virulence of *B. anthracis*. Although heme is toxic to all bacterial pathogens [36], *B. anthracis* can grow on a high concentration of heme relative to other bacilli [37], and regulates its resistance to heme toxicity during infection through the HssRS two-component system [38]. Therefore, investigating the regulation of the *B. anthracis asb* and *bac* siderophore operons when heme is used as an iron source will also be an important avenue for future work.

In summary, we have demonstrated that the biosynthesis of petrobactin and bacillibactin in *B. anthracis* is subject to repression by increasing iron concentration, but that petrobactin biosynthesis is less affected by this level of regulation than bacillibactin. Whereas the transcription and biosynthesis of bacillibactin is effectively abrogated in the presence of even low levels of iron, petrobactin accumulation occurs in medium containing up to 20 µM iron, and transcription of the petrobactin biosynthetic operon can be detected even in iron-rich conditions. In addition, the presence of oxygen appears to support more active transcription of the *asb* operon and results in higher levels of end-point petrobactin accumulation. Conversely, the highest concentration of bacillibactin isolated so far was observed in iron-depleted conditions under low oxygen tension. Hence, the differences between petrobactin and bacillibactin regulation suggest that *B. anthracis* has evolved multiple strategies for metal acquisition.

## Materials and Methods

### Siderophores

Authentic siderophore standards were obtained as iron-free compounds. The catecholate siderophores petrobactin and bacillibactin were isolated from culture supernatants of the *B. anthracis* strain Sterne 34F_2_ in Iron Depleted Medium (IDM) [11] as described previously [12], [15]. Petrobactin and bacillibactin were dissolved in water and methanol, respectively, as stock solutions in this study.

### Bacterial Strains

Bacterial strains used for this study are summarized in Table 5. All *B. anthracis* strains were derived from the wild type strain Sterne 34F_2_ [39], and were propagated at 37°C in LB or LB + kanamycin (40 µg/ml) as necessary.

**Table 5:** Bacterial strains used in this study.

Strain	Relevant characteristics	Reference
*Bacillus anthracis*		
Sterne 34F_2_	Wild type (pXO1^+^, pXO2^−^)	35
KDC3	34F_2_, Δ*sodA1*::Km^r^	14
KDC5	34F_2_, Δ*sodA2*	14
KDC7	34F_2_, Δ*dhbACEBF*	15
BA850	34F_2_, Δ*asbABCDEF*	12

### Bacterial Growth Conditions for Isolation of Petrobactin and Bacillibactin in IDM with Iron and Paraquat Stress and High and Low Aeration

For IDM cultures under iron variation: starter cultures of *B. anthracis* Sterne 34F_2_ and mutants *ΔsodA1*, *ΔdhbACEBF*, and *ΔasbABCDEF* were grown overnight in Brain-Heart Infusion (BHI) medium at 37°C with shaking at 300 rpm. Cells were spun down by centrifugation and washed three times in IDM. Cells were back-diluted into IDM to an OD_600_ of 0.10 into IDM with 0, 3, 6, 10, and 20 µM FeSO_4_ • 7H_2_O. Cultures of the strains *ΔdhbACEBF* and *ΔasbABCDEF* were grown only with two concentrations of 0 and 20 µM FeSO_4_ • 7H_2_O as controls. Cultures were shaken at 300 rpm at 37°C for 24 hours and then filtered in 0.02 µM pore-size Corning filter flasks. For IDM cultures with paraquat treatment: starter cultures of *B. anthracis* Sterne 34F_2_ and *ΔsodA1* were grown as described above. Cultures were shaken at 300 rpm at 37°C for 24 hours with 0, 5, 15, 25 and 50 µM paraquat and filtered as described above. For high and low aeration cultures with varying iron: approximately 10^5^ spores of *B. anthracis* Sterne 34F_2_ or *ΔsodA1* were added to 50 ml of IDM with 0, 3, 6, 10 and 20 µM FeSO_4_ • 7H_2_O. High aeration cultures were shaken at 300 rpm in plastic baffled 500 ml volume flasks (10% v/v ratio). Low aeration cultures were shaken at 110 rpm in plastic 125 ml volume flasks (40% v/v ratio). Cells were incubated for 24 hours at 37°C, and a final OD_600_ was taken. Cultures were then filtrated as stated above. All filtrated supernatants were used for siderophore analysis.

### Bacterial Growth Conditions for RNA Isolation

RNA was collected from *B. anthracis* Sterne 34F_2_ grown in IDM with either 0 (-Fe) or 20 (+Fe) µM FeSO_4_ • 7H_2_O added to the medium. Cells grown in “rich” medium were grown in LB broth. Starter cultures were grown overnight, and back-diluted 1:20. Cells were harvested by centrifugation when they reached OD_600_ ∼0.4 – 0.6 (mid log phase) for RNA collection. High aeration cells were grown 10% v/v in plastic baffled 500 ml flasks (50 ml) at 37°C and shaken at 300 rpm. At the target OD_600_, 10 ml of cells were harvested and pelleted by centrifugation at 4000 rpm at 5°C for 10 minutes. Low aeration cells were grown in a 40% v/v plastic 125 ml flask (50 ml) at 37°C and shaken at 110 rpm. RNA was collected using the Ambion Ribopure Bacteria Kit with modifications [15]. RNA preparations were cleaned up using the Qiagen RNEasy Cleanup Protocol with an on-column DNase digestion. RNA concentration was calculated using the A_260/280_ method on a Beckman Spectrophotometer, and RNA integrity was checked on the Agilent Bioanalyzer system.

### Quantification of Siderophore Petrobactin and Bacillibactin

For quantification of petrobactin: Filtrated supernatants (10 ml) from IDM cultures were adjusted to pH 7.0. Two grams of Amberlite® XAD-16 resin (Supelco, PA) was added into the supernatant and the mixtures were shaken for one hour at 150 rpm in the dark. Mixtures were then filtered, and resin washed with pure water three times and eluted with 10 ml of methanol. Methanol eluates were concentrated to dryness *in vacuo* and dissolved in 100 µl of 50% methanol for quantification. For quantification of bacillibactin: IDM cultures (10 ml) were adjusted to pH 2.0 and then extracted with equal volumes of ethyl acetate three times. The organic layers were pooled and the solvents were concentrated into dryness *in vacuo*. The dried pellets were dissolved in 100 µl of methanol and used for quantitative analysis of bacillibactin.

The XAD-16 resin- or organic solvent extracts from the culture supernatants of *B. anthracis* were analyzed for quantification of petrobactin and bacillibactin, respectively, using a Shimadzu LCMS-2010EV system. (Detailed description on LCMS analysis can be found below). The 3,4-dihydroxybenzoic acid (for petrobactin) and 2,3-dihydroxybenzoic acid (for bacillibactin) were utilized as internal standards for signal normalization. 10 µl of a 3,4-dihydroxybenzoic acid or 2,3-dihydroxybenzoic acid stock solution (1 mM each) was added to the 100 µl of each sample. With the LC methods applied below, petrobactin and 3,4-dihydroxybenzoic acid showed retention times of 15.989–16.289 and 18.133 min and bacillibactin and 2,3-dihydroxybenzoic acid showed retention times of 19.204–19.300 and 17.367 min, respectively. The calibration curves were constructed by using a series of ten concentrations (ranging from 10 to 500 µM) of authentic petrobactin and bacillibactin for quantification. The retention time and mass spectrum of the corresponding petrobactin or bacillibactin peaks in each sample were compared with those of authentic petrobactin and bacillibactin standards. The petrobactin and bacillibactin quantities were calculated in µg/ml culture supernatant. Quantifications were performed three times from the independent extraction, and the mean values and standard deviations were calculated from the total data set.

### LCMS Analysis

LC was performed on a Shimadzu LC-20AD HPLC system consisting of a UV/VIS detector (SDP-20AV) and an autosampler (SIL-20AC). The HPLC was coupled to a Shimadzu LCMS-2010EV mass spectrometer with an electrospray ionization (ESI) interface. LC was carried out on an analytical column (Waters XBridge® C_18_, 3.5 µm, 2.1 ×150 mm) using a linear stepwise gradient from 5% to 50% aqueous acetonitrile in 0.1% (v/v) formic acid (FA) at a flow rate of 0.1 ml/min over 30 minutes for petrobactin analysis. The solvent systems with a gradient from 10% to 100% aqueous acetonitrile in 0.1% (v/v) FA over 30 minutes were used for bacillibactin analysis under the same LC conditions as described above. The ESI source was set at the positive mode. Selected ion monitoring (SIM) was conducted to monitor ions at *m/z* 719.3, 883.2, and 155.2, which corresponded to the protonated molecular ions of petrobactin, bacillibactin, and internal standards 3,4-dihydroxybenzoic acid and 2,3-dihydroxybenzoic acid, respectively. The MS operating conditions were optimized as drying gas 1.5 l/min, CDL temperature 250°C, heat block temperature 200°C, and detector voltage 1.5 kV.

### SYBR-Green Quantitative RT-PCR

The gene *fusA*, which encodes a constitutively transcribed elongation factor (elongation factor G), was used as a standard for normalization of transcript from each of two experiments. The 700 ng of the RNA preps were used to make cDNA using 300 ng of random primers and Invitrogen SuperScript II Reverse Transcriptase, following the maker's protocol with an overnight incubation at 42°C. For all samples, a no RT control was performed. Diagnostic PCR of the cDNA was done using 1.5 µl of each cDNA prep to eliminate the possibility of genomic DNA contamination (all negative), and for the confirmation of DNA synthesis using primers for the *fusA* gene. SYBR green PCR was performed on an ABI Prism 7900 HT at the University of Michigan cDNA Affymetrix Core at the Comprehensive Cancer Center. Applied Biosystems SYBR Green Master Mix was used with 1.5 µl of the cDNA preparations. Each reaction was performed 2 times and each primer set was run with (i) cDNA, (ii) the cDNA made with no RT control, and (iii) with no cDNA. The 40 elongation cycles were performed with 30-second elongation times and an annealing temperature of 55°C (optimized beforehand), with 94°C melting temperature and 68°C extension temperature. Primers used were for an internal segment of the second gene in the *bac* operon (*dhbC*, GBAA2369), an internal segment of the second gene in the *asb* operon (*asbB*, GBAA1982), and internal segments of the *sodA1* and *sodA2* genes (all PCR products were between 180 and 200 base pairs) (Table 6). Threshold (C_T_) values are the values where the PCR enters exponential amplification. C_T_ values used were the mean of the results of 4 reactions (2 replicates per 2 biological replicates). Approximate transcript abundance was analyzed using the Comparative C_T_ Method (or 2^–[delta][delta]Ct^ Method). The *fusA* control product C_T_ value is subtracted from each of the experimental C_T_ values. The difference in transcript abundance is then calculated as 2^-(difference in normalized CT's)^.

**Table 6:** Oligonucleotide primers used in this study.

Gene name and number^a^	Nucleotide sequence (5′-3′ direction)
*dhbC* (GBAA2369)	TCTTCGAGAATTAGCAGAGCA
	TCCTCCGCTCTTCTTTTATCT
*asbB* (GBAA1982)	TCGAGCGTAATGTATGATACGAA
	TCGTAGCCAATTCTCTATCCCAT
*fusA* (GBAA0107)	AGGTCACGTAGATTTCACAGT
	GAGTATAAGAAATCTGCACCG
*sodA1* (GBAA4499)	TAAGATTGTCCAGAAGAATGT
	CATACAAAACACCATAACACG
*odA2* (GBAA5696)	CCTAATGGAGACGTTGCAAAA
	GCAAAGGTGTATCTTGATTCG

a GBAA numbers are from the Ames Ancestor genome contained in the TIGR Comprehensive Microbial Resource (http://cmr.tigr.org/tigr-scripts/CMR/CmrHomePage.cgi).

### End-Point RT-PCR

700 ng of RNA collected as stated above was used to perform endpoint RT-PCR using Invitrogen one-step RT-PCR with Platinum *Taq* according to the manufacturer's instructions. Briefly, RT was performed at 50°C for 30 minutes. PCR was performed with 0.25 pg of operon/gene-specific primers for 30 cycles with an elongation temperature of 70°C and an extension time of 30 seconds. 5 µl of endpoint PCR product was run on 0.7% agarose gels and visualized by ethidium bromide staining. Negative controls omitting reverse transcriptase and positive controls with *B. anthracis* Sterne 34F_2_ genomic DNA were done with each experiment. PCR products were designed to result in ∼200 base pair products.

### SOD Enzymatic Assays from Bacterial Cell Lysates

SOD enzymatic activity from whole cell lysates of *B. anthracis* Sterne 34F_2_ and *ΔsodA1* was measured by the Nitroblue Tetrazolium Salt (NBT) assay, modified from Oberly and Spitz [40]. Lysates were collected as follows: *B. anthracis* Sterne 34F_2_ cultures were grown in LB medium with or without 1 hour treatment with 800 µM paraquat, or in IDM with high and low aeration (with and without 20 µM FeSO_4_ • 7H_2_O supplementation) as described above. At mid-exponential phase (or after treatment with paraquat), cells were pelleted by centrifugation at 4°C, washed two times in ice cold PBS, and resuspended in 500 µl of 0.05M potassium phosphate buffer (pH 7.8) with 20 mM benzamidine. Cells were lysed via bead-beating with acid washed glass beads (diameter, 150–200 µm; Sigma G1145) for 8 1-minute cycles set on homogenize with resting on ice for at least 2 minutes between pulses (Biospec mini-bead beater). Tubes were spun at 1,000 rpm at 4°C for 10 minutes. Supernatants were transferred to fresh tubes and spun at 4°C for 10 minutes at 5,000 rpm. Supernatants were transferred to fresh tubes and spun at 4°C at 14,000 rpm for 45 minutes and final supernatants were transferred to fresh tubes. Total protein was quantified with the Qubit Quant-iT Protein Assay (Invitrogen/MolecularProbes). Modified NBT assay is as follows: 800 µl of SOD reaction buffer (1 mM diethylenetriaminepenta-acetic acid (DETAPAC, Sigma D6518) in 0.05 M potassium phosphate buffer (pH 7.8); 1 unit of catalase (Sigma C8531); 5.6×10^−5^ M NBT (Sigma N5514); 10^−4^ M xanthine (Sigma X0626)) and 200 µl of 0.05 M potassium phosphate buffer were added to a 1ml plastic cuvette and the solution was blanked on a Beckman DU530 spectrophotometer. Then, ∼60 mU of xanthine oxidase (from bovine milk, Sigma X4500) suspended in 0.05 M potassium phosphate buffer (pH 7.8) was added to the cuvette, and absorbance at 560 nm (A_560_) was read every 30 seconds for 3.5 minutes. The rate of increase of absorbance was recorded and used as the control rate (optimized to be between 0.20 and 0.25 A/min). Next, whole cell lysates in a 200 µl volume with 1, 3, 5, or 10 µg of protein were added to 800 µl SOD reaction buffer and blanked. A ∼60 mU of xanthine oxidase was added and the increase of A_560_ was measured as with the control. Percent inhibition was calculated as: % inhibition  =  [(control rate – sample rate) / control rate] ×100. At least 3, and not more than 6, biological replicates were assayed, and final figures are the mean of the biological replicates with standard deviations.
